# Antidiabetic efficacy of lactoferrin in type 2 diabetic pediatrics; controlling impact on PPAR-γ, SIRT-1, and TLR4 downstream signaling pathway

**DOI:** 10.1186/s13098-018-0390-x

**Published:** 2018-12-04

**Authors:** Waleed A. Mohamed, Mona F. Schaalan

**Affiliations:** 1 Department of Chemistry, Kasr El Aini Teaching Hospitals, Cairo University, Cairo, Egypt; 2 Department of Clinical Pharmacy and Pharmacy Practice, Translational Research Unit, Faculty of Pharmacy, Misr International University, Cairo, Egypt

**Keywords:** Lactoferrin, T2DM, PPAR-γ, SIRT-1, NFκB

## Abstract

The current study aims to investigate the antidiabetic efficacy of camel milk-derived lactoferrin and potential involvement of PPAR-γ and SIRT-1 via TLR-4/NFκB signaling pathway in obese diabetic pediatric population. Sixty young obese patients with type 2 diabetes were selected from the Pediatric Endocrine Metabolic Unit, Cairo University and were randomly divided among two age and sex-matched groups so as to receive either standard therapy without lactoferrin in one arm or to be treated with oral lactoferrin capsules (250 mg/day, p.o) for 3 months in the other arm. Both groups were compared to 50 control healthy volunteers. Measurements of HbA1c, lipid profile, antioxidant capacity (SOD, Nrf2), proinflammatory interleukins; (IL-1β, IL-6, IL-18), Cyclin D-1, lipocalin-2, and PPAR-γ expression levels were done at the beginning and 3 months after daily consumption of lactoferrin. The mechanistic involvement of TLR4-SIRT-1-NFκB signaling cascade was also investigated. The antidiabetic efficacy of lactoferrin was confirmed by significant improvement of the baseline levels of HbA1c, BMI and lipid profile of the obese pediatric cohort, which is evidenced by increased PPAR-γ and SIRT-1 expression. Moreover, the anti-inflammatory effect was evident by the significant decrease in serum levels of IL-1β, IL-6, IL-18, TNF-α, lipocalin 2 in type 2 diabetic post-treatment group, which corresponded by decreased NFκB downstream signaling indicators. The antioxidant efficacy was evident by stimulated SOD levels and NrF2 expression; compared with the pre-treatment group (all at *P* ≤ 0.001). The consumption of high concentrations of lactoferrin explains its hypoglycemic efficacy and counts for its insulin-sensitizing, anti-inflammatory and immunomodulatory effects via TLR4-NFκB-SIRT-1 signaling cascade. Recommendations on regular intake of lactoferrin could ensure better glycemic control, compared to conventional antidiabetics alone.

## Introduction

Despite established therapies for type 2 diabetes mellitus (T2DM), the occurrence of insulin resistance and glucose level fluctuations remains a challenge [1]. The uncontrolled hyperglycemia and induced metabolic disorders will ultimately lead to micro- and macrovascular complications [2]. Type 2 diabetes is thought to involve chronic inflammation, which is manifested by the production of different inflammatory mediators. Several proinflammatory cytokines were shown to be elevated in serum of diabetic patients with a new onset of diabetes or a longstanding disease [3]. Furthermore, cytokines have been implicated in the development of the insulin resistance, metabolic syndrome and micro/macrovascular complications of T2DM [4]. Multiple studies have suggested the potential involvement of proinflammatory interleukin-1 (IL-1) pathway in β-cell destruction. [5, 6]. IL-1, alone or combined with other proinflammatory cytokines such as IFN-β, can cause β-cell destruction in islets via pathways involving mitogen-activated protein kinase and nuclear factor κB. (NF-κB) [7–9]

IL-6 is a pleiotropic cytokine with a major role as immunoregulator and non-immune cell types and tissues as fibroblasts, endothelial cells, monocytes/macrophages, and a variety of tumor cell lines [10]. IL-18 is one of the mediators of innate immunity initiated by host–pathogen interaction, which is mainly produced by monocytes/macrophages in response to stimuli of viral/bacterial origin [11].

Camel milk has been gaining intense interest due to a myriad of therapeutic properties it exerts in various pathologies, such as anticarcinogenic, antimicrobial, antioxidant, hypocholesterolemic, as well as antidiabetic effects. Concerning the reported hypoglycemic effect of camel milk, it is attributed to the high amount of insulin as well as the presence of bioactive compounds in milk [12–16], like lactoferrin, which is  deemed responsible for most of its therapeutic effects.

Lactoferrin (Lf) is an iron-binding glycoprotein from the transferrin family that has pleiotropic biological and immunological activities including antimicrobial, immunomodulatory, and antineoplastic properties. Many of these functions do not appear to be connected with its iron binding ability, though Lf is a member of a transferrin family [5, 17].

Lactoferrin concentration is 230 times higher in camel milk vs that of the bovine source [7, 18], which could rationalize the difference in biological activity of camel milk. However, the relationship between its concentration and physiological or pathological effects on body functions is not yet well characterized, to enable the full and safe utilization of this glycoprotein. Lactoferrin consists of two equal halves, designated as N-lobe and C-lobe, each of which contains one iron-binding site. While the N-lobe of lactoferrin is known for its enhanced antimicrobial effect, the C-lobe of lactoferrin mediates various therapeutic functions which are still being discovered. The potential of the C-lobe in the treatment of diabetes has been previously indicated, but the exact mechanism not thoroughly elucidated [19].

During an infection or an inflammatory condition, the levels of Lf are raised in the body [20] making Lf a biomarker for inflammatory conditions. Lf has been found to have a therapeutic potential and for this reason, there have been several attempts to isolate Lf from various sources. Antimicrobial and anti-inflammatory activity reports on Lf identified its significance in host defense against infection and extreme inflammation.

Furthermore, several studies reported that Lf regulates inflammatory cytokines production in a mode resembling other anti-inflammatory cytokines by suppressing inflammation interacting with macrophages and restraining the production of inflammatory cytokines by cells [21]. Lf is known to suppress the production of TNF-α, IL-1β, IL-6, and IL-8 in human mononuclear cells (in vitro*)* and improve production of IL-10 and IL-4 (in vivo) [22] which explain its ability to reduce β-cell destruction; however, the down stream regulators are still obscure.

Highlighting the role of Lf in the activation of immune cells, it enters in the intestinal microvilli via receptors of lactoferrin and transferrin on brush border membranes from human intestine that are present on the mucosal surface of the intestinal cells [23].

The absorption and the transportation of intestinally administrated bovine lactoferrin (LF) were immunohistochemically and physiochemically investigated in the small intestine, as per Kitagawa et al. [24]. At the apical halves of the small intestinal villi, bovine LF was absorbed by transcytosis as small vesicles through villous columnar epithelial cells. This suggests that the absorption was mediated by LF-binding factors on the epithelial cell membranes. Almost all of the absorbed bovine LF was demonstrated to be transported via the lymphatics as well as the portal vein into the systemic circulation [24].

The lactoferrin molecule further boosts up the immune response due release of IFN-γ, TNF-α, IL-6 as well as activation of NK cells, PMNs and CD3+ and CD4+ T cells. Finally, the lactoferrin enters the cells by receptor-mediated endocytosis where it is released within the cells once the receptors are digested by endosomes.

Toll-like receptors (TLRs) are a class of pattern recognition receptors of the innate immune system. They mainly function for initiating an inflammatory cascade in response to the recognition of danger-associated molecular patterns. Among the 13 known TLR subtypes, TLR-4 downward signaling causes the production of pro-inflammatory cytokines. Induction and persistence of inflammation that is associated with the progression of diabetic nephropathy are previously demonstrated to be mediated through TLR-4. TLR-4 signaling activates nuclear factor kappa B (NF-κB), that translocate into the nucleus, thus, activating a set of inflammatory genes producing IL-1β, IL-6 and TNFα [25]. Moreover, TLR-4 allows activation of p38MAPK, further mediating the NF-κB hyperinflammatory responses [26].

In diabetic patients, hemoglobin A1c (HbA1c) levels are responsible for stimulating TLR-4 expression in monocytes suggesting a molecular link between inflammation and metabolic control in the metabolic syndrome-associated disorders such as hyperglycemia, dyslipidemia, and hemodynamic abnormalities, implicated to represent a predisposing factor for diabetes and potential complications [27].

Sirtuin 1 (SIRT-1), the mammalian homolog of yeast silent information regulator 2 (Sir2), is an NAD^+^-dependent histone deacetylase and an important coordinator of the mammalian metabolic response to fasting and caloric restriction [28]. During periods of nutrient deprivation, elevated levels of SIRT-1 in the liver increase hepatic glucose production [29] and induce the expression of oxidative machinery.

As new therapeutic targets aim is to improve insulin resistance and prevent the short/long-term complications of type 2 DM, the current research aims to investigate the potential contribution of peroxisome proliferator-activated receptor-γ (PPARγ), Cyclin-D-1 and lipocalin in the pathophysiology of disease progression and potential efficacy of camel milk on these markers.

Peroxisome proliferator-activated receptor-γ (PPARγ) is a nuclear hormone receptor that maintains homeostasis of glucose by activating glucokinase and glucose transporter 2 (GLUT2) in the liver and pancreas. In addition, PPARγ has insulin-sensitizing effects in peripheral tissues as well as the ability to sense blood glucose in pancreatic β-cells [30, 31]. In this regard, the researchers were investigating antidiabetic drugs/nutraceuticals by exerting effects via PPARγ.

Cyclin-D-1, an important G1 component, is responsible for adaptive proliferative responses of β-cells to insulin resistance. Genetic lineage tracing experiments in β-cells suggest that proliferation is the major factor in the maintenance of β-cells in vivo and that pancreatic β-cell mass results from a dynamic balance of neogenesis, proliferation, cell size, and apoptosis [32]. The role of cyclins D, as G1 components, in β-cells was demonstrated by reduced β-cell mass and hyperglycemia in mice deficient for these G1 components [33]. This implies that deregulation of cell cycle components such as Cyclin D-1 could be an important component for adaptive proliferative responses of β-cells to insulin resistance.

Lipocalin 2 (LCN2) or neutrophil gelatinase-associated lipocalin is abundantly produced by adipocytes. It was originally identified as a 25-kDa protein produced from human neutrophils and belongs to the superfamily of lipocalins [34]. The expression and secretion of LCN2 increase sharply after the conversion of preadipocytes to mature adipocytes. Its expression can be induced by different inflammatory stimuli, including lipopolysaccharide and IL-1β [35, 36].

As new therapeutic targets aim is to improve insulin resistance and prevent the short/long-term complications of type 2 DM, the current research aims to investigate potential therapeutic benefits of oral Lf on glycemic control, through assessment of cytokines, inflammatory status, immunomodulatory effects in children with type 2 diabetes. The current clinical study also addresses the possible contribution of lactoferrin-induced expression of PPAR-γ expression, hence amelioration of the diabetic profile in pediatrics via TLR4, SIRT-1, and NFκB signaling pathway.

## Materials and methods

### Subjects

This prospective, randomized clinical study included 60 children (mean age; 14 ± 3 years) diagnosed with type 2 diabetes who were randomly recruited from the Diabetic Endocrine Pediatric outpatient clinics, Alzeraeyeen Hospital, Giza, Egypt. Patients were not allowed to take any concomitant medicine throughout the study period, except their standard antidiabetic therapies.

A detailed history was taken from the parents, and all the patients and control subjects underwent complete medical examination, anthropometric measurements (body weight, height) as well as pubertal evaluation by the same pediatric endocrinologist. A testicular volume equal to or greater than 4 mL in boys and the onset of breast development in girls were accepted as the criteria for the onset of puberty. The recruited sixty young obese patients with type 2 diabetes were randomly divided among two age and sex-matched groups so as to receive either standard therapy without lactoferrin in one arm or to be treated with LF capsules (Jarrow Formulas^®^, USA, 250 mg/day, p.o) for 3 months in the other arm. The tested lactoferrin was intended to be derived from camel milk colostrums, therefore requested a special formula of Jarrow Formulas^®^ derived from the colostrums of camel milk. Monitoring of potential side effects was also considered and reported. Both groups were compared to 50 control healthy volunteers.

According to the American Academy of Pediatrics (AAP), the management of type 2 diabetes in children and adolescents should begin with lifestyle modification and metformin treatment. The goal of therapy is to achieve and maintain euglycemia, as well as near-normal hemoglobin A_1c_ (HbA_1c_) levels (≤ 7%). If metformin is unsuccessful as monotherapy, the addition of insulin, a sulfonylurea, or another hypoglycemic agent may be appropriate. Hemoglobin A1c (HbA_1c_) levels should be measured every 3 months and treatment adjusted if goals for both HbA_1c_ and blood glucose are not met.

Exclusion criteria included all patients with any complications like hypoglycemia, ketoacidosis, and cardiovascular event, renal or acute infection was not included in the study. The control group consisted of 50 age and sex-matched individuals recruited from the outpatient clinic. All subjects gave informed consent after explanation of the study aim and the nature of lactoferrin as a natural protein derived from the colostrums of camel milk. The study followed the principles of the Declaration of Helsinki and was approved by the local Ethics Committee of El Ziraeyeen Hospital, Giza-Egypt.

### Blood sampling

Fasting venous blood samples were collected (≈ 2 mL/subject) and divided into two portions; the first was collected for separation of sera, while the other was immediately transferred into Ficoll reagents for isolation of monocytes and generation of cell lysis products for determination of cyclin D-1, NF-κB and TLR-4. Moreover, their nuclear extracts were used for the determination of PPAR-γ, NrF2 and SIRT-1 levels. Separation of sera was done by centrifugation (3500×*g*, 10 min) and all sera were stored at − 70 °C until analyses. The resultant sera samples were analyzed to determine the levels of glucose, insulin, lipid profile, and IL-1β, IL-6, IL-18, Lipocalin, and SOD activity. Analysis of glycosylated hemoglobin (HbA1c), lipid profile, superoxide dismutase, lipocalin 2, IL-1β, IL-6, and IL-18 were measured at the beginning and 3 months after consumption of Lf daily.

### Determination of serum glucose (FBG), insulin and lipid profile

Fasting blood glucose (FBG) was measured by using the hexokinase method, and fasting insulin (FINS) was measured by ELISA kit (Bio Source International Inc., Europe S.A.). Serum total cholesterol (TC), triglyceride (TG), high-density lipoprotein cholesterol (HDL-C) were measured enzymatically (Stanbio, North Main Street, Boerne, Texas, USA) in all samples. Low-density lipoprotein cholesterol (LDL-C) was calculated using the Friedewald equation; LDL-C = TC − TG/5 − HDL-C. Intra-assay and Inter-assay coefficients of variations were respectively 2.1% and 1.9% for FBS, 1.0% and 1.4% for TC, 3.4% and 3.3% for TG, 0.8% and 1.3% for LDL, 4.5% and 3.7% for HDL, and 4.9% and 4.2% for insulin. Insulin resistance was calculated by the homeostasis model assessment of insulin resistance (HOMA-IR) equation: HOMA-IR = [FBS (mg/dL) × fasting insulin concentration (µU/L)/22.5] [37–39].

### Determination of plasma lactoferrin (Lf)

Plasma Lf concentration was assessed in plasma using an ELISA kit (Assaypro EL2011-1, St.-Charles, USA) that is highly specific to human Lactoferrin. This assay employed a quantitative sandwich enzyme immunoassay technique, which measured LF in 5 h. A monoclonal antibody specific for human LF had been pre-coated onto a microplate. Human LF in standards and samples was sandwiched by the immobilized antibody and biotinylated polyclonal antibody specific for human LF, which was recognized by a streptavidin-peroxidase conjugate. All unbound material was then washed and a peroxidase enzyme–substrate was added. The color development was stopped and the intensity of the color was measured. The intra- and inter-assay coefficients of variation were 4.8 and 7.3%, respectively.

### Determination of serum IL-1 IL-6, IL-18, SOD, lipocalin 2

IL-1β and IL-6, measured by using commercial kit; Human IL-1β ELISA Kit and IL-6 Human IL-6 ELISA kits, respectively, were from Anogen-YES Biotech Laboratories Ltd, Ontario, Canada. Assay ranges were; 2–400 pg/mL and 7–2000 pg/mL, respectively. IL-18 was measured using a commercial ELISA kit (CUSABIO Company, China). The IL-18 level is expressed in pg/mL, and the detection range is 31.25–2000 pg/mL. Determination of serum lipocalin 2 level was assayed by lipocalin-2/NGAL Human ELISA kit (BioVendor Research and Diagnostic Products, Czech Republic) and its limit of detection is 0.02 ng/mL. Human Superoxide dismutase was measured using commercial ELISA kit (Kamiya biomedical company, Seattle, USA).

### Isolation of peripheral blood monocytes, and lysate preparation

Blood was suspended over a layer of Ficoll reagent and centrifuged for 20 min at 1800×*g* and room temperature to separate peripheral blood mononuclear cells. These cells were then transferred to a fresh tube and washed twice with phosphate-buffered saline (PBS, pH 7.4), and then re-suspended in 500 µL ice-cold Cell Lysate Buffer (50 mM Tris, pH 7.4, 250 mM NaCl 1 mM Na_3_VO_4_, 5 mM EDTA 0.02% NaN_3_) supplemented with protease and phosphatase inhibitors. The lysates were generated by re-suspended by repeat-pipetting and incubation of the materials at 2–8 °C for 30 min. After this period, the lysed cells were centrifuged (13,000 rpm, 10 min, 2–8 °C) and the resulting supernatants were transferred to clean tubes. These lysates were either then used immediately or sub-aliquoted and stored at − 70 °C until use for the determination of cyclin D-1, NF-κB and TLR-4.

### PBMC nuclear extract isolations

An aliquot of the lysed cells was used for isolation of nuclear fraction using Nuclear Extract commercial Kit (Active Motif, Carlsbad, CA, USA). The nuclear fractions were stored at − 80 °C until the assessment of Nrf2, PPAR-γ, and SIRT-1.

### Determination of cyclin D-1, TLR4, and NF-κB in PBM-lysate samples

Cyclin D1 was assessed using an ELISA kit, Lifespan Biosciences Inc, Seattle, USA. The detection range was 0.313–30 ng/mL, Sensitivity: Typically less than 0.119 ng/mL and Intra-Assay CV (< 10%); Inter-Assay CV (< 12%). The ELISA method was adopted to test the protein concentration of TLR4, NF-κB and the test was carried out according to the manufacturer’s instruction (Germany, IBL company).

### Determination of SIRT-1, NrF2, and PPAR-γ in PBM-nuclear extract samples

Nuclear extracts were used to verify the protein contents of NF-κB p65 using Transcription Factor Assay Kit (Colorimetric) (ab210613; Abcam, MA 02139-1517 USA). This assay is a semiquantitative, high throughput assay to quantify NF-κB p65 activation in nuclear extracts. This assay combines a quick ELISA format with a sensitive and specific non-radioactive assay for transcription factor activation. Detection limit: < 0.5 µg nuclear extract/well, and detection range: 0.2–10 µg nuclear or whole cell extract/well.

PPAR-γ was assessed using ELISA assay kit (MyBiosource, San Diego, CA, USA), detection range (0.156–10 ng/mL) and sensitivity; 0.094 ng/mL.

SIRT-1 was assayed using Human SIRT ELISA kit (ab171573), sensitivity; 132 pg/mL and testing range 0.63–40 ng/mL. Intra-assay CV; 2%, and Inter-assay; 4.1%.

### Statistical analysis

Data are represented as mean ± SD for quantitative data, and as frequency and percentage for qualitative data. Differences between groups were assessed by one-way analysis of variance (ANOVA), followed by a Bonferroni post hoc multiple comparison tests, including diabetic group before and after LF treatment. A P-value of less than or equal to 0.05 was considered statistically significant using SPSS for Windows, version 15.0 software was used for statistical analysis.

## Results

The mean age of diabetic pediatric groups and their control cohort was 14 ± 3 and 13 ± 4 years, respectively (difference found was statistically non-significant). Male to female ratio in diabetic patients was 34:26 (Table 1). We were first interested to compare the Lf concentration in all studied groups.

**Table 1:** Glycolytic and lipid profile in children with type 2 diabetes before and after lactoferrin treatment

Variables	Diabetic patients (N = 30)		Controls (N = 30)
	Before lactoferrin	After lactoferrin	
Age	12–17		11 ± 17
Gender			
Male/female	34/26		28/22
BMI (kg/m^2^)	30.9 ± 2.5^a^	28.8 ± 2.1	21.2 ± 1.2
Lactoferrin (ng/mL)			
Range	25.6–35.5	44.2–60.5	9.7–19.1
Mean ± SD	29.73 ± 4.05^a^	52.35 ± 6.75^b^	14.01 ± 3.00
FPG (mg/dL)			
Range	197–269^a^	131–189^b^	67–110
Mean ± SD	233 ± 36	130 ± 29	87 ± 22
HbA1c (%)	9.8 ± 1.3^a^	7.2 ± 1^b^	5.3 ± 1.5
Insulin range	2.87–14.4^a^	24.5–35.6^b^	7.3–10.3
Total cholesterol (mg/dL)	240.5 ± 13.2^a^	170.6 ± 11.2^b^	110.67 ± 9.2
Triglycerides (mg/dL)	183.4 ± 6.5^a^	131 ± 10.2^b^	75.23 ± 0.92
HDL-C (mg/dL)	27.4 ± 3.5^a^	38.5 ± 3.3^b^	42.87 ± 3.2
LDL-C (mg/dL)	176.5 ± 12.5^a^	105.9 ± 8.2^b^	53.2 ± 3.2

Values are means ± SDM

*BMI* body mass index, *FPG* fasting plasma glucose, *HbA1c* glycated hemoglobin

^a^Significant difference from normal group, at P ≤ 0.05

^b^Significant difference from diabetic patients before therapy, at P ≤ 0.05

Interestingly, the compensatory twofold significant increase of Lf content was evident in the diabetic pediatrics compared to their control counterparts. The supplementation of Lf increased its level further to 44.20–60.50 ng/mL, with a mean ± SD (52.35 ± 6.75 ng/mL). Table 1 presents the glycolytic and lipid profile before and after LF consumption, where it depicts the ameliorative effect of LF consumption on glycolytic metabolism; in terms of significant reduction of FBG (33.5%) and HbA1c (25.7%), but increase in insulin (eight fold). Concerning the effect of LF on BMI, it showed nonsignificant change before and after LF consumption. Concerning total cholesterol levels, after LF consumption, it was significantly decreased (28.35%), compared to the levels before Lf consumption. This effect was in alignment with the reduction of TG (31.7%) and LDL-C (37%), as well as an increase in HDL-C (17.7%).

To unveil the mechanistic pathway behind the antidiabetic and anti-inflammatory, hence antidiabetic efficacy, we first determined the levels of TLR4 protein contents in control and T2DM subjects. Then, we measured the downstream functional readouts (IL-1β, IL-6, IL-8, TNF-α) of NF-κB. There was a significant up-regulation of proinflammatory cytokines; IL-1β, IL-6, IL-18, and lipocalin 2 (4-, 3.4-, 4- and 3-fold increase, respectively) in T2DM patients compared with control group, as illustrated in Table 2. Upon consumption of Lf, these elevated markers were significantly decreased (24.3%, 31.5%, 34.6%, 35%, respectively), compared with pre-treatment group.

**Table 2:** Effect of lactoferrin on the serum levels of SOD, IL-1β, -6, -18 and lipocalin in type 2 diabetic patients

Variables	Diabetic patients (N = 30)		Controls (N = 30)
	Before lactoferrin	After lactoferrin	
SOD (U/mL)	143.6 ± 9.344^a^	175.9 ± 10.3^b^	222.5 ± 14.9
Interleukin-1β (pg/mL)	13.8 ± 1.8^a^	10.45 ± 1.37^b^	3.4 ± 0.8
Interleukin-6 (pg/mL)	16.78 ± 2^a^	11.5 ± 1.89^b^	4.9 ± 1.0
Interleukin-18 (pg/mL)	46.9 ± 6.4^a^	30.67 ± 4.15^b^	15.5 ± 2.87
Lipocalin 2 (pg/mL)	102.2 ± 11.7^a^	66.36 ± 13.2^b^	25.6 ± 3.9

Values are means ± SDM

*SOD* superoxide dismutase, *IL* interleukin

^a^Significant difference from normal group, at P ≤ 0.05

^b^Significant difference from diabetic patients before therapy, at P ≤ 0.05

**Fig. 1:**
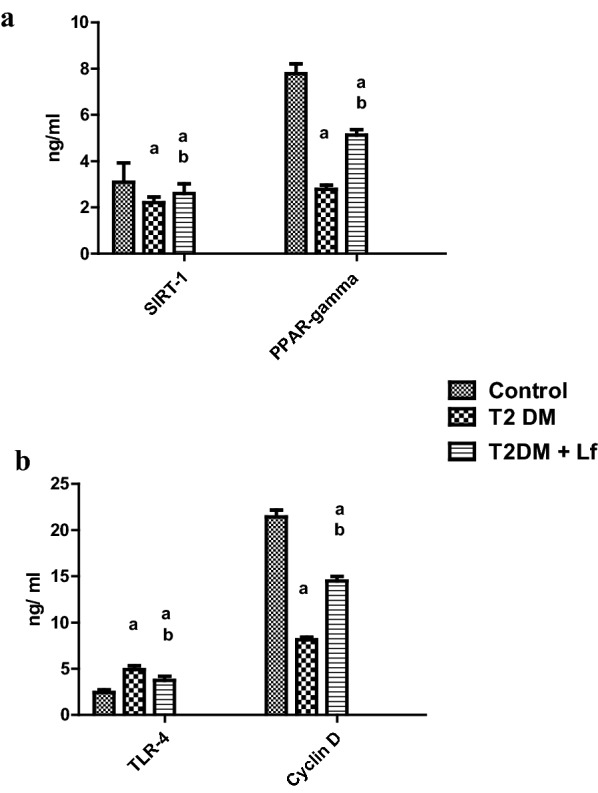
Protein levels of SIRT-1, PPAR- γ (**a**), TLR-4, Cyclin D-1 (**b**) in diabetic pediatrics with/without lactofrrin treatment, compared to the control cohorts. *Significant difference from control group at P < 0.05. ^Ψ^Significant difference from Lf/insulin group at P < 0.05

In terms of nuclear extract of PBMs, we assessed PPAR-γ and SIRT-1, which were significantly decreased in the diabetic group and ameliorated in the LF group (Fig. 1a).

The PBMs levels of TLR-4 of diabetic groups were significantly increased (two fold, Fig. 1b), compared to control levels, and upon consumption of Lf significantly ameliorated. On the other hand, Cyclin D levels in the diabetic groups were significantly decreased (62%, Fig. 1b), compared to control levels, and upon consumption of Lf significantly ameliorated. (Table 3, P ≤ 0.001).

**Table 3:** Effect of lactoferrin on the PBM levels of Cyclin D-1, PPAR- γ, SIRT-1, TLR-4, and NFκB in type 2 diabetic patients before and after lactoferrin treatment

Variables	Diabetic patients (N = 30)		Controls (N = 30)
	Before lactoferrin	After lactoferrin	
PBM lysate			
TLR-4 (ng/mL)	4.9 ± 1.0^a^	3.76 ± 1.07^b^	2.43 ± 0.69
NFκB-p65 (µg/mL)	183.20 ± 49.19^a^	98.68 ± 29.80^b^	15.5 ± 2.87
Cyclin D-1 (ng/mL)	8.13 ± 0.66^a^	14.49 ± 1.2^b^	21.43 ± 1.82
PBM nuclear extract			
PPAR-γ (ng/mL)	2.78 ± 0.44^a^	5.13 ± 0.56^b^	7.79 ± 1.04
SIRT-1 (ng/mL)	2.2 ± 0.6^a^	2.6 ± 1.03^b^	3.1 ± 2.04
NrF2/ARE binding activity	0.06 ± 0.01^a^	0.08 ± 0.01^b^	0.12 ± 0.069

Values are means ± SDM

^a^Significant difference from normal group, at P ≤ 0.05

^b^Significant difference from diabetic patients before therapy, at P ≤ 0.05

**Fig. 2:**
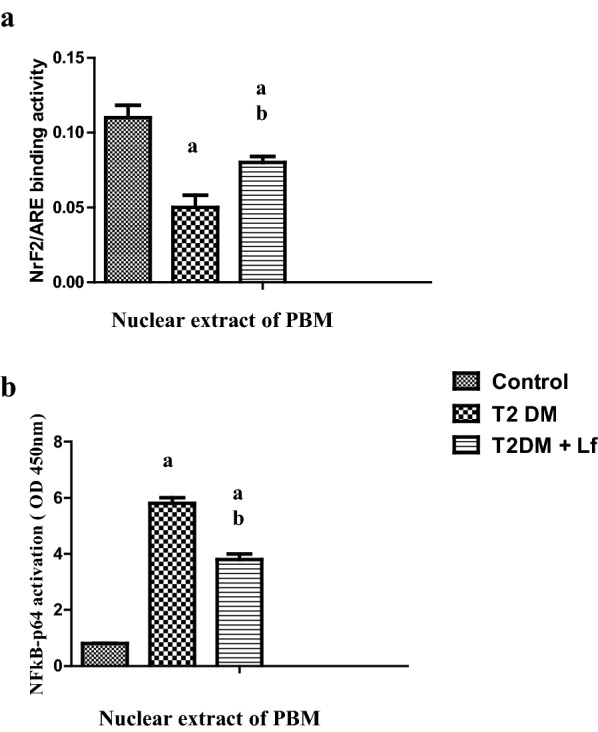
Protein levels of NrF2/ARE (**a**), NFkB (**b**) in diabetic pediatrics with/without lactofrrin treatment, compared to the control cohorts

As for the antioxidant parameters SOD and NrF2 (Fig. 2a), both were significantly increased in the Lf supplemented group, in alignment with a boosted expression of NF-kB (Fig. 2b), compared to the diabetic group without LF.

Concerning the potential side effects of Lf, the monitoring sheets and reports indicated were diarrhea (11%), loss of appetite (2%), fatigue (5%), and constipation (6%) have been reported.

## Discussion

This is the first study that reports a compensatory increase in circulating Lf in association with improvement in glycemic and metabolic parameters in a pediatric obese cohort of type 2 diabetes, as presented in Table 1. Moreover, we proved that the antidiabetic efficacy of Lf was mechanistically evidenced by amelioration of the glycolytic homeostasis, dyslipidemic profile, and anti-inflammatory clinical outcomes. Similar antidiabetic efficacy of Lf, a major active component in camel milk, has been reported in many clinical and experimental studies [40, 41], but the exact pathway was not thoroughly elucidated.

**Fig. 3:**
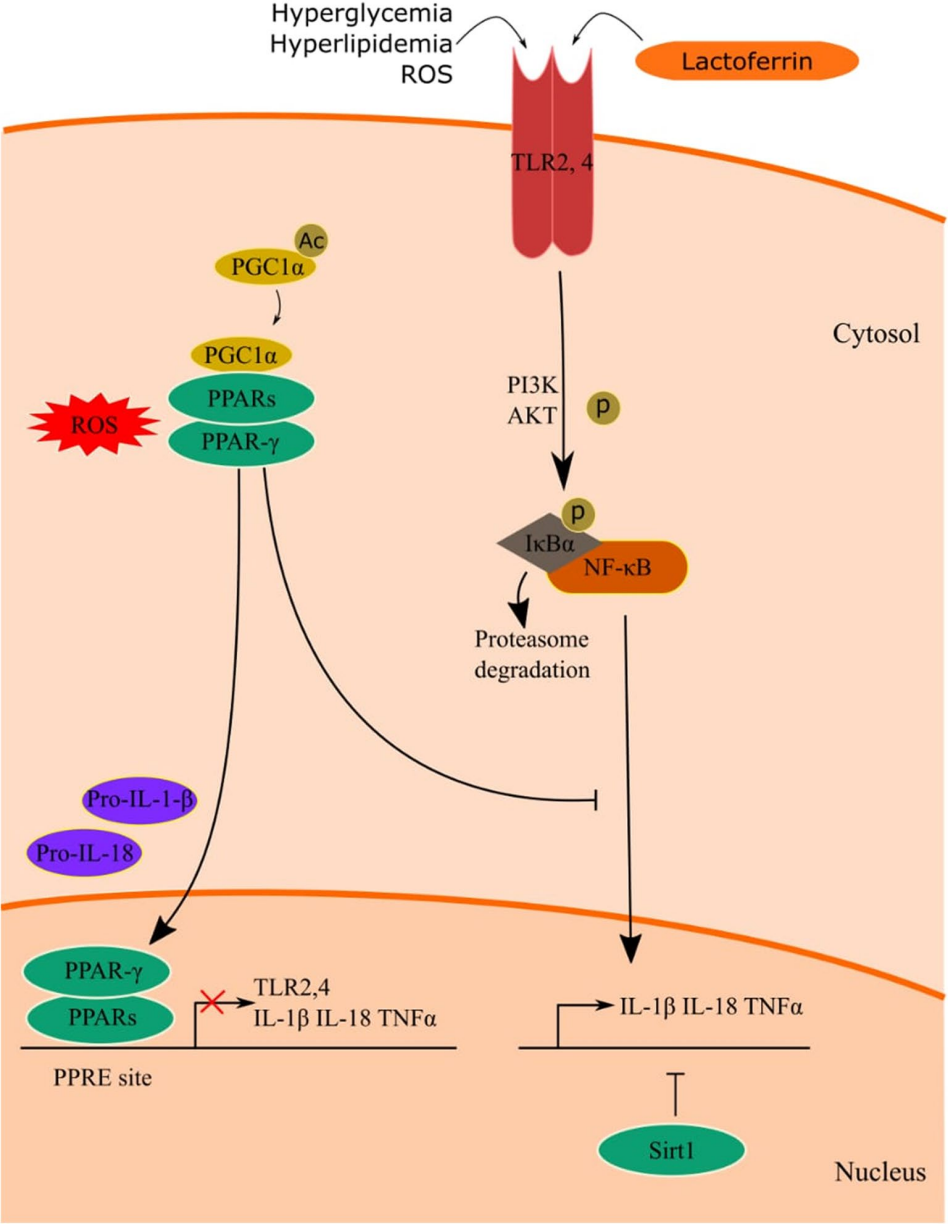
The proposed crosstalk between surface TLRs and dysregulated metabolic factors in diabetes, by interfering with NF-κB signaling pathway, and induced proinflammatory nuclear gene overexpression; e.g.IL-1β, IL-18, and TNF-α. Moreover, the figure illustrates the targeting of lactoferrin on PPAR-γ (cytoplasmic and nuclear) and SIRT-1 (intranuclear) via a controlling impact on TLR4 downstream NF-κB signaling pathway

Investigating the mechanism behind the antidiabetic effects of Lf, we were able to demonstrate that the hypoglycemic, hypolipidemic and associated anti-inflammatory effects have been modulated via the TLR-4, NF-κB, SIRT-1 axis, an important signaling pathway known to activate transcription regulators of inflammation. The proposed crosstalk between TLRs and dysregulated metabolic factors in diabetes and potential targets of Lactoferrin in the impediment of this vicious paradigm is illustrated in Fig. 3. Lactoferrin-induced activation of PPAR-γ and SIRT-1 may control TLR pathways by interfering with proinflammatory gene overexpression, in an NF-κB signaling pathway [42].

In the current study, Lf significantly abrogated the TLR-4 induced NF-κB stimulation, which leads to dropping off the proinflammatory cytokine release of IL-1β, IL-6, IL-18, TNF-α, lipocalin 2 in type 2 diabetic post-treatment group. These promising effects were correlated to the PPAR-γ/SIRT-1 stimulating effects.

The association of the hyperglycemic milieu and the induced chronic inflammation, as an immunological response in type 2 diabetes, has been previously reported in several experimental and clinical studies. The adipocyte/peripheral inflammatory cells produce various proinflammatory cytokines associated with the inflammatory response (insulitis) within the islets described as pancreatic β-cell toxicity [5].

At the immunological level, Lf is reported to activate the immune response in vitro at the site of injury and accumulates the neutrophils which activate the phagocytic activity of polymorphonuclear leukocytes (PMNs) and macrophages. As a result, the pro-inflammatory cytokines decrease in number and the activity of natural killer (NK) cells increases, supporting, thereby, the activation of lymphocytes. Using two known signaling pathways, nuclear factor-kappa B (NF-κB) and MAPkinase, Lf at the cellular level modulates the differentiation, maturation, activation, migration, proliferation, and functions of immune cells [43]. However, the current study focused on the NF-κB signaling pathway of Lf.

Separate from all the body fluids, the iron-free form of Lf is stored in the cytoplasmic secondary granules of neutrophils, but during inflammation is increasingly released at the site of inflammation at very high concentration, playing a major role in the feedback mechanism of the inflammatory response [44]. This rationalizes, why Lf is reported to play a vital role as a mediator of systemic inflammatory response syndrome by allowing the controlled regulation of inflammation without any pathological damage [45].

The herein reported boosted antioxidant activity of SOD and NrF2 expression, exerted by Lf supplementation was also shown to support the immune response via antioxidant mechanism [46]. Experimental studies in cells demonstrated that lactoferrin significantly inhibited/decreased intracellular reactive oxygen species (ROS) levels in a dose-dependent manner and protected from oxidative stress. As the lipolytic induction of FFA increases the oxidative stress, the putative decrease of FFA induced by Lf could also contribute to its antioxidant efficacy [47].

A significant decrease of Nrf2 in T2DM pediatric patients was reported in the current study, followed by a subsequent elevation upon Lf treatment. Lower levels of Nrf2 in individuals with poor glucose control are previously reported, but the exact mechanism by which hyperglycemia causes a decrease in the activation of Nrf2 is unknown in peripheral blood mononuclear cells. Some studies in podocytes and cardiomyocytes, incubated with high glucose concentrations, show that Nrf2 transcript is increased [48, 49]. However, we assume that most severe hyperglycemic states have a longer negative effect on the induction of Nrf2, by yet unknown mechanisms, coupled with an inflammatory condition that may influence the activation of Nrf2.

Toll-like receptors are known to represent basic elements in the innate immune system involved in the progression of the proinflammatory signaling pathways. Thus, inhibition of TLR-4 could be a potential therapeutic target in diabetes and its potential vascular complications through suppressing inflammation and interstitial macrophage infiltration. These effects are likely mediated through the inhibition of NF-κB activation, a master switch of inflammation in diabetic microvascular pathologies [26, 27].

The current study shows that Lf decreased diabetes-induced inflammation by inhibiting the TLR-4-NF-κB axis with subsequent reduction in serum proinflammatory cytokines; IL-1β, IL-6, and IL-18, in diabetic children. Furthermore, the amelioration in pro-inflammatory cytokines was accompanied by a significant reduction in lipocalin-2. This was further supported by the positive alignment demonstrated hereby between the levels of lipocalin-2, IL-8, and the investigated pro-inflammatory cytokines as well as TLR-4 protein level. These results are in accord with a previous study reporting that pharmacological inhibition of TLR-4 conferred vasculoprotection in diabetic mice [27].

The Lf-induced decrease in TLR-4 level, in the current study, could be attributed to the fact that Lf has anti-inflammatory as well as anti-oxidative properties [50, 51], which was evident by the restoration of SOD and NrF2 levels towards normal values, with a statistically significant difference from the control group. The Lf-induced modulation of the TLR-4 pathway and the pro-inflammatory cascade, via inhibition of reactive oxygen species generation and through the direct use of anti-inflammatory agents, was previously reported [52]. Similarly, and at the cellular level, Lf was shown to modulate differentiation, activation, migration, proliferation, and functions of immune cells using the signaling pathways, NF-κB and MAPK [53].

It has been previously reported that hyperglycemia-induced TLR-4 stimulation creates an inflammatory milieu, and causes recruitment of monocytes, hence stimulates IL-6 secretion [54]. This rationalized why diabetic patients have elevated blood levels of IL-6, which, together with TNF-α are known to increase a chronic state of inflammation and the development of vascular disease, possibly by increasing oxidative stress [55]. IL-1 is believed to cause transient insulinopenic diabetes, therefore using the IL-1 blockers could protect from both types of diabetes [56]. IL-6 is also a proinflammatory cytokine, which promotes inflammation and adaptive immune responses [57]. In patients with T2DM, IL-6 levels were found to be significantly increased in the group with long T2DM duration, as well as in those newly diagnosed, when compared with healthy controls, with the highest levels in the newly diagnosed patients [58]. IL-18 belongs to the IL-1 superfamily of cytokines. In alignment with IL-12, IL-18 stimulates polarization of Th1 cells, hence the activity of NK cells which augments IFN-γ production [59].

Interestingly, we observed that the anti-inflammatory effect of Lf takes place by suppression of TNF-α and IL-1β. The inhibitory effect of Lf on proinflammatory cytokines suggested that Lf, released from secondary granules of activated neutrophils at an inflammatory site, may provide an inhibitory feedback mechanism to prevent excessive neutrophil aggregation and activation in colitis experiments [60, 61].

In addition, Lf was shown to lower inflammation, oxidative stress (OxS) and apoptosis, which are key mechanisms involved in the progression of various cardiometabolic disorders [12–14], while few reports have emphasized its anti-adipogenic actions [62, 63].

Currently, adipocytes are not conceptualized as fat reservoir any more, but active cells secreting numerous adipokines and chemokines, that work together to regulate inflammation, insulin sensitivity/resistance, and glucose homeostasis systemically and peripherally [64]. LCN2 was recently added to the myriad of secreted adipocytokines that regulate glycolytic metabolism [65]. Our results revealed increased levels of LCN 2 in diabetic patients who are significantly improved by Lf intake. The correlation of LCN2 with the lipid profile revealed significant, positive association.

In the setting of dominating inflammatory medium of diabetes, the expression, and secretion of LCN2 increases stridently due to the conversion of preadipocytes to mature adipocytes. The expression of the latter can be induced by different inflammatory stimuli, including lipopolysaccharide and IL-1β. Therefore, recent therapies target the reduction of the uncontrolled cytokines production and novel therapeutic targets to effectively inhibit subsequent tissue damage [66].

As for the mechanistic pathways of Lf-induced hypoglycemia, the current research is the first to test the association between Lf and PPAR-γ and the potential association between Cyclin D-1 and PPAR-γ. The current study highlights a significant reduction of PPAR-γ and Cyclin-D1 levels in type 2 diabetic patients, an effect that was successfully reversed of camel milk colustrum containing Lf. This should highlight the relation of Cyclin D-1 and glycemic control that camel milk Lf is reported to execute. To the best of the authors’ knowledge, this is among very few clinical studies to investigate the role of Cyclin-D beyond oncologic disorders. A recent study of Hosooka and Ogawa [67] on cyclin D-1 clarified its relation to the physiologic and pathologic glucose metabolism. They explained that cyclin D1 is a regulator of the cell cycle that promotes the transition from G1 to S phase by activating cyclin-dependent kinase 4 (CDK4) or CDK6. Albeit the reported role of cyclin D-1 in hepatic damage, regeneration, or carcinogenesis, Bhalla et al. [68] recently highlighted that cyclin D-1 represses hepatic gluconeogenesis and oxidative phosphorylation through inhibition of PGC1a activity in a CDK4-dependent mechanism.

Results of the current research revealed a clear association between Lf-induced hypoglycemic effect and increase in PPAR-γ and Cyclin D-1 expressions. This indicates that Lf does exert not only hypoglycemic effects, due to its insulin-like effect, but also via its insulin-sensitizing efficacy, that is postulated to be through the PPAR-γ-dependent pathway. The latter is a nuclear hormone receptor that maintains homeostasis of glucose by activating rate-limiting enzyme of glycolysis, glucokinase, as well stimulating the hepatic glucose uptake, via glucose transporter 2 (GLUT2), in the liver and pancreas. In addition, PPARγ has insulin-sensitizing effects in peripheral tissues as well as the ability to sense blood glucose in pancreatic β-cells [69, 70]. As pancreatic β-cell failure is a major component of both types of diabetes, as well as insulin resistance [1], modulators of cycle components such as Cyclin D-1, could play an important role for adaptive proliferative responses of β-cells to insulin resistance [71].

Lactoferrin also directly interacted with modified LDL to prevent its interaction with scavenger receptors. A region rich in basic amino acid residues near the lactoferrin N terminus is responsible for the interaction with acetylated or oxidized LDL. This cationic part of Lf strongly binds modified LDLs via electrostatic interaction with positively charged Arg residues at physiological pHs [72]. The mechanism of Lf action might involve several processes, such as inhibition of adipogenesis, a decrease of dietary triglyceride absorption, elevation of HDL cholesterol possessing anti-atherogenic properties, inhibition of accumulation of oxidized LDL cholesterol forms in macrophages and protection against the formation of foam cells [73].

In previous studies, it was shown that circulating Lf concentration was inversely associated with fasting triglyceride concentration, body mass index (BMI), waist-to-hip ratio, and fasting glucose concentration, and directly correlated with HDL cholesterol concentration (r = 0.21; P = 0.004) [63, 72].

To the best of the authors’ knowledge, the current study is the first to investigate the potential contribution of PPAR-γ, SIRT-1 and pathways to the anti-inflammatory and immunomodulatory functions of the Lf on pediatrics with type 2 diabetes via possible involvement TLR4, Cyclin D-1, and NFκB signaling pathway. The dose was chosen based on previous clinical trials which tested Lf in clinical and experimental diabetes, human cancers as well as cancer cell lines [74]. Although these results are promising, larger studies are warranted. To this end, we conclude that elevation of serum Lf concentration may play an important role in returning the immunity balance to the prime state by its action in recurrence of immunity of diabetic patients especially the vascular dysfunction which represents the main problem of Egyptian diabetic patients. Detection of Lf concentration may act as new parameters to evaluate the sequelae of diabetes especially concerning the major and minor vasculopathy in Egyptian diabetic patients. It is also recommended to use Lf as a promising, completely non-toxic, natural remedy which might be applied in long-term prophylaxis and therapy of metabolic disturbances, such as insulin resistance/type II diabetes.

## Abbreviations

IDDM: insulin dependent diabetes mellitus
LCN2: lipocalin 2
p-Akt: phosphorylated Akt
PI3K: phosphoinositide 3-kinase
PPAR-γ: peroxisome proliferator-activated receptor gamma
SIRT-1: sirtuin-1, silent receptor-1
SOD: superoxide dismutase
T2DM: type2 diabetes mellitus
TAC: total antioxidant capacity
TLR-4: toll-like receptor-4
